# Development of a Metric to Detect and Decrease Low-Value Prescribing in Older Adults

**DOI:** 10.1001/jamanetworkopen.2021.48599

**Published:** 2022-02-15

**Authors:** Thomas R. Radomski, Alison Decker, Dmitry Khodyakov, Carolyn T. Thorpe, Joseph T. Hanlon, Mark S. Roberts, Michael J. Fine, Walid F. Gellad

**Affiliations:** 1 Division of General Internal Medicine, Department of Medicine, University of Pittsburgh School of Medicine, Pittsburgh, Pennsylvania; 2 Center for Pharmaceutical Policy and Prescribing, Health Policy Institute, University of Pittsburgh, Pittsburgh, Pennsylvania; 3 Center for Health Equity Research and Promotion (CHERP), Veterans Affairs (VA) Pittsburgh Healthcare System, Pittsburgh, Pennsylvania; 4 RAND Corporation, Pardee RAND Graduate School, Santa Monica, California; 5 Division of Pharmaceutical Outcomes and Policy, Eshelman School of Pharmacy, University of North Carolina at Chapel Hill, Chapel Hill; 6 Division of Geriatric Medicine, University of Pittsburgh School of Medicine, Pittsburgh, Pennsylvania; 7 Geriatric Research Education and Clinical Center, VA Pittsburgh Healthcare System, Pittsburgh, Pennsylvania; 8 Department of Health Policy and Management, University of Pittsburgh Graduate School of Public Health, Pittsburgh, Pennsylvania

## Abstract

**Importance:**

Metrics that detect low-value care in common forms of health care data, such as administrative claims or electronic health records, primarily focus on tests and procedures but not on medications, representing a major gap in the ability to systematically measure low-value prescribing.

**Objective:**

To develop a scalable and broadly applicable metric that contains a set of quality indicators (EVOLV-Rx) for use in health care data to detect and reduce low-value prescribing among older adults and that is informed by diverse stakeholders’ perspectives.

**Design, Setting, and Participants:**

This qualitative study used an online modified-Delphi method to convene an expert panel of 15 physicians and pharmacists. This panel, comprising clinicians, health system leaders, and researchers, was tasked with rating and discussing candidate low-value prescribing practices that were derived from medication safety criteria; peer-reviewed literature; and qualitative studies of patient, caregiver, and physician perspectives. The RAND ExpertLens online platform was used to conduct the activities of the panel. The panelists were engaged for 3 rounds between January 1 and March 31, 2021.

**Main Outcomes and Measures:**

Panelists used a 9-point Likert scale to rate and then discuss the scientific validity and clinical usefulness of the criteria to detect low-value prescribing practices. Candidate low-value prescribing practices were rated as follows: 1 to 3, indicating low validity or usefulness; 3.5 to 6, uncertain validity or usefulness; and 6.5 to 9, high validity or usefulness. Agreement among panelists and the degree of scientific validity and clinical usefulness were assessed using the RAND/UCLA (University of California, Los Angeles) Appropriateness Method.

**Results:**

Of the 527 low-value prescribing recommendations identified, 27 discrete candidate low-value prescribing practices were considered for inclusion in EVOLV-Rx. After round 1, 18 candidate practices were rated by the panel as having high scientific validity and clinical usefulness (scores of ≥6.5). After round 2 panel deliberations, the criteria to detect 19 candidate practices were revised. After round 3, 18 candidate practices met the inclusion criteria, receiving final median scores of 6.5 or higher for both scientific validity and clinical usefulness. Of those practices that were not included in the final version of EVOLV-Rx, 3 received high scientific validity (scores ≥6.5) but uncertain clinical usefulness (scores <6.5) ratings, whereas 6 received uncertain scientific validity rating (scores <6.5).

**Conclusions and Relevance:**

This study culminated in the development of EVOLV-Rx and involved a panel of experts who identified the 18 most salient low-value prescribing practices in the care of older adults. Applying EVOLV-Rx may enhance the detection of low-value prescribing practices, reduce polypharmacy, and enable older adults to receive high-value care across the full spectrum of health services.

## Key Points

**Question:**

Can a scalable and broadly applicable metric be developed using common forms of health care data to detect low-value prescribing among older adults?

**Findings:**

In this qualitative study of 27 low-value prescribing practices, a panel of experts was convened using an online modified-Delphi approach to develop a scientifically valid and clinically useful low-value prescribing metric. This panel identified the 18 most salient low-value prescribing practices, which were included as a component of the final metric.

**Meaning:**

The metric developed in this study may enhance the detection of low-value prescribing practices, reduce polypharmacy, and enable older adults to receive high-value care.

## Teaser

This qualitative study describes the process of building EVOLV-Rx (Evaluating Opportunities to Decrease Low-Value Prescribing) with a panel of clinicians, deprescribing experts, health care leaders, and other stakeholders.

## Introduction

Low-value care, which is defined as the use of health services whose harms or costs exceed their benefits, is a major factor in wasteful health care spending and has been associated with physical, psychological, and financial harms.^1,2^ In the US, more than $100 billion per year has been spent on the delivery of low-value care, affecting up to 43% of Medicare beneficiaries.^1,3^ However, low-value care metrics primarily consist of tests and procedures and exclude the low-value prescribing of medications. This exclusion represents a major gap in the ability to systematically detect low-value prescribing and low-value care across the full spectrum of health services.^3,4^

Characterizing and reducing low-value prescribing are especially important for individuals who are 65 years or older. More than 40% of older adults have been subjected to polypharmacy (prescribed ≥5 medications), and 30% to 50% have been prescribed a potentially inappropriate medication, increasing their risk for adverse drug events and hospitalizations.^5,6^ In addition, 35% of Medicare Part D beneficiaries have experienced hardship in paying for their medications.^6,7^

There is currently no metric that consolidates and prioritizes the hundreds of recommendations from professional societies and the Choosing Wisely campaign for health systems and third-party payers to detect, quantify, and reduce low-value prescribing.^8^ Many current recommendations, such as avoiding the use of testosterone to treat nonspecific symptoms of aging, provide only general guidance to clinicians and have not been operationalized to measure low-value prescribing using administrative claims or electronic medical record data, which would ensure the scalability and automation of these measures.^9^ Other tools, such as the Beers Criteria,^10^ may be applied to health care data but focus primarily on medication safety and do not incorporate other components of health care value, such as cost. The perspectives of patients or their caregivers were not integral to the development of these and other low-value care metrics.^9^

In this qualitative study, we aimed to develop a scalable and broadly applicable metric that contains a set of quality indicators to detect and reduce low-value prescribing among older adults and that is informed by diverse stakeholders’ perspectives. We named this metric EVOLV-Rx (Evaluating Opportunities to Decrease Low-Value Prescribing).

## Methods

**Figure 1.**
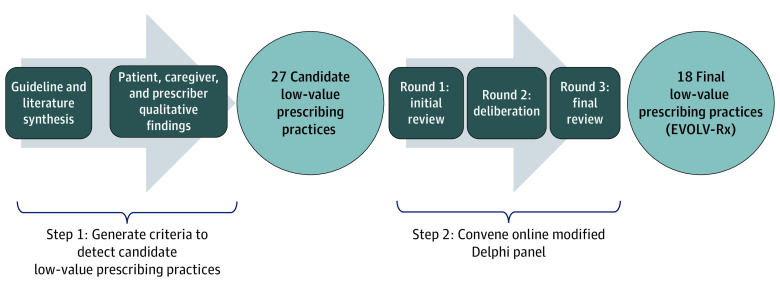
The Development of Evaluating Opportunities to Decrease Low-Value Prescribing (EVOLV-Rx)

We developed EVOLV-Rx in 2 sequential steps. First, we generated the criteria to detect candidate low-value prescribing practices. For this step, we synthesized the preexisting recommendations from the Choosing Wisely campaign and other medication safety criteria (eg, the Beers Criteria), peer-reviewed literature, and the results of 3 qualitative studies on low-value prescribing.^11,12,13^ Second, using an online modified-Delphi approach, we convened a panel of physicians and pharmacists to codify the final components of EVOLV-Rx (Figure 1). Members of this panel serve as health system leaders, practicing clinicians, and researchers. This study was deemed exempt by the institutional review boards of the University of Pittsburgh and the RAND Corporation.

### Step 1: Generating the Criteria to Detect Candidate Low-Value Prescribing Practices

We compiled a collection of low-value prescribing recommendations according to previously applied approaches to identifying low-value health services.^3,14^ We considered prescribing practices whose costs or harms generally outweigh their benefits for older adults (aged ≥65 years). Practices were assessed on the following 4 value domains, which were adapted from the Lown Institute approach to characterizing medication appropriateness^15^: (1) lack of effectiveness for a stated indication, either in general or because of inappropriately prolonged use; (2) potential for harm because of the risk of an adverse drug event, drug-drug or drug-disease interaction, or overly intensive treatment; (3) excessive cost because of the unnecessary use of a costly brand-name medication or specific preparation; or (4) use of a medication to treat the adverse effects of another medication as part of a prescribing cascade.

We acquired low-value prescribing recommendations from the Choosing Wisely campaign^8^ and the most up-to-date versions of the following medication safety and appropriateness guidelines: the Beers Criteria,^10^ FORTA (Fit for the Aged) list,^16^ STOPP/START (Screening Tool of Older Persons’ Prescriptions and Screening Tool to Alert to Right Treatment),^17^ and STOPPFrail (Screening Tool of Older Persons’ Prescriptions in Frail Adults With Limited Life Expectancy).^18^ We also considered the findings from published studies and medications or medication classes that were most associated with adverse drug events requiring hospital admission.^19^ We integrated the findings from 3 qualitative studies (which were conducted by some of us and other members of our research team), which used focus groups with patients and caregivers as well as semistructured interviews with primary care physicians to characterize their perspectives on and specific examples of low-value prescribing (eTable 1 in the Supplement).^11,12,13^

To focus on those candidate low-value prescribing practices that were current and had the potential to affect the greatest number of older adults, we narrowed down our initial collection of low-value prescribing recommendations to those that overlapped with the 100 most frequently prescribed or costly medications among Medicare beneficiaries in fiscal year 2017 (the most recent year of available data at the beginning of this study). We also included medications whose frequency of use was comparable to drugs that were most frequently prescribed in Medicare Part D but may be purchased over the counter, such as aspirin or docusate sodium.^20^

All of the investigators on the research team met monthly to generate operational definitions that could be applied to health care data for each candidate low-value prescribing practice. An external advisory panel of 6 geriatricians, pharmacoepidemiologists, and medication safety experts also met every 3 months to provide general feedback and ensure that EVOLV-Rx remained focused on the prescribing practices that were most relevant to older adults.

To define each candidate low-value prescribing practice, we developed both sensitive and specific criteria, applying an approach developed by Schwartz et al.^3,21^ The goal of the sensitive criteria was to identify patients who could be subjected to potential low-value prescribing, whereas the goal of the specific criteria was to identify the subset of older patients for whom the prescribing practice would most likely be of low value, according to a priori low-value criteria we had established. For example, regarding the prolonged use of proton pump inhibitors (PPIs), the proposed sensitive criteria may identify patients who were prescribed a PPI for more than 2 months. Recognizing that the prolonged use of a PPI may at times be clinically appropriate, the specific criteria would build on the sensitive criteria to recognize situations in which PPI use would most likely be of low value, such as among patients without a guideline-concordant indication, without concurrent long-term use of NSAIDs and corticosteroids, or with a prescription for a brand-name PPI.

To facilitate the deliberations of the online modified-Delphi panel, we generated a 2-page peer-reviewed infographic for each candidate low-value prescribing practice. The infographic contained the proposed sensitive and specific criteria as well as an evidence-based synopsis and related utilization, cost, and qualitative data (3 examples are provided in the eFigure in the Supplement). Before implementing them in the study, we received feedback on the criteria for each candidate low-value prescribing practice as well as on the accuracy and content of the infographic from a multidisciplinary clinical faculty that was affiliated with the Center for Pharmaceutical Policy and Prescribing at the University of Pittsburgh.

### Step 2: Convening an Expert Panel

To establish consensus on and refine the criteria to identify the most salient low-value prescribing practices for EVOLV-Rx, we convened a panel of 15 physicians and pharmacists using an online modified-Delphi method. This panel comprised practicing clinicians; health system leaders; and international experts in deprescribing, pharmacoepidemiology, and health care value (Box). Panelists were recruited on the basis of their record of scholarly publications, academic or industry leadership, and referral by members of the external advisory panel or research team. To conduct the activities of the panel, we applied a validated, 3-round approach using ExpertLens software package (RAND).^22,23,24,25^ Before its use in this study, the data collection protocol using ExpertLens was tested by 3 local clinicians.

Box. **Online Modified-Delphi Panel of Experts**

Panelists included members of the external advisory panel and were recruited on the basis of their record of scholarly publications, academic or industry leadership, or referral by the advisory panel or research team.

**Member Specialties, Affiliations, and Geographic Locations**

Tim Anderson, MD, MAS, Beth Israel Deaconess Medical Center, Harvard Medical School, Boston, Massachusetts^a,d^
Elizabeth Bayliss, MD, MSPH, University of Colorado School of Medicine and Kaiser Permanente Colorado, Aurora, Colorado^c,d^
Winfred Frazier, MD, MPH, New Kensington Family Health Center, University of Pittsburgh Medical Center, Pittsburgh, Pennsylvania^c,f^
Mark Friedberg, MD, MPP, Blue Cross Blue Shield of Massachusetts, Boston, Massachusetts^a,e^
Chester B. Good, MD, MPH, Center for Value-Based Pharmacy Initiatives, UPMC Health Plan, Pittsburgh, Pennsylvania^a,e^
Holly Holmes, MD, MS, University of Texas McGovern Medical School, Houston, Texas^b,d^
Matthew Joseph, MD, PharmD, Northern Medical Associates, University of Pittsburgh Medical Center, Pittsburgh, Pennsylvania^a,f^
Zach Marcum, PharmD, PhD, University of Washington School of Pharmacy, Seattle, Washington^d^
Chris Moriates, MD, University of Texas at Austin Dell Medical School, Austin, Texas^a,d^
Natasha Parekh, MD, MS, Queens Health System, Honolulu, Hawaii^ a,e^
Emily Reeve, BPharm, PhD, University of South Australia, Adelaide, South Australia, Australia^d^
Gordon Schiff, MD, Brigham and Women's Hospital, Harvard Medical School, Boston, Massachusetts^a,d^
Kenneth Schmader, MD, VA Durham Healthcare System, Duke University School of Medicine, Durham, North Carolina^b,d^
Michael Steinman, MD, VA San Francisco Healthcare System, University of California, San Francisco, San Francisco, California^b,d^
Justin Turner, BPharm, MClinPharm, PhD, Canadian Deprescribing Network, Centre de Recherche de l'Institut Universitaire de Gériatrie de Montréal, Québec, Canada^d^

^a^
General internal medicine specialty.

^b^
Geriatrics specialty.

^c^
Family medicine specialty.

^d^
Academia affiliation.

^e^
Payer organization affiliation.

^f^
Community practice affiliation.

The panel was engaged for 3 rounds between January 1 and March 31, 2021, and each round lasted 1 to 2 weeks. In round 1, the panelists rated the scientific validity and clinical usefulness of the criteria to detect each candidate low-value prescribing practice using a 9-point Likert scale (median score ranges: 1 to 3 indicating low validity or usefulness; 3.5 to 6, uncertain validity or usefulness; and 6.5 to 9, high validity or usefulness^26^) and provided corresponding comments to explain their ratings (eTable 2 in the Supplement provides a full description of the panel questions and rating interpretation). In round 2, the panelists reviewed each other’s numeric and free-text responses for each candidate low-value prescribing practice, as depicted by descriptive statistics, statements about the existence of agreement among panelists, and bar charts that were automatically generated by ExpertLens. Next, the panelists participated in an anonymous and asynchronous online discussion, which one of us moderated (T.R.R.). Informed by the round 1 findings and round 2 discussion, we revised or clarified the proposed sensitive and specific criteria. In round 3, informed by the scores and deliberations in rounds 1 and 2, the panelists provided their final ratings for each candidate low-value prescribing practice. In each round, individual members of the panel reviewed the practices in random order to ensure that robust ratings were given across all candidate low-value prescribing practices.

### Statistical Analysis

We applied the RAND/UCLA Appropriateness Method to panelist scores in rounds 1 and 3 to ascertain whether there was agreement among the panelists and to identify the degree of scientific validity and clinical usefulness for each candidate low-value prescribing practice.^26^ This validated method has been used to achieve consensus on the content of clinical guidelines and quality metrics in an online modified-Delphi process.^26^

Using the RAND/UCLA Appropriateness Method, we looked for the presence of agreement by first calculating the interpercentile range (IPR) between the 70th and 30th percentiles of panelist scores. Next, we calculated the IPR adjusted for symmetry with the following equation: IPRAS = 2.35 + (AI × 1.5), where AI represented the asymmetry index, which is defined as the distance between the central point of the IPR and 5, the central point of the 9-point Likert scale used by panelists to rate each candidate low-value prescribing criteria. If the IPR was greater than the IPR adjusted for symmetry, then there was no agreement. If the IPR was less than the IPR adjusted for symmetry, then there was agreement. For the low-value prescribing criteria wherein agreement was found, we characterized their scientific validity and clinical usefulness according to the median scores. The final metric included only candidate low-value prescribing practices that showed panel agreement and received median scores of 6.5 or higher, which indicated both high scientific validity and high clinical usefulness.

## Results

### Low-Value Prescribing Practices

A total of 527 low-value prescribing recommendations were compiled from the Choosing Wisely campaign, other medication safety criteria, and peer-reviewed literature, along with an additional 101 medications or medication classes that were cited as being potential low-value prescribing practices by the participants of the qualitative studies we examined (eTable 1 in the Supplement). Of these medications, 63 overlapped with the costliest or most frequently prescribed medications among Medicare beneficiaries in 2017. We further consolidated medications from the same class or category into a single operationalized definition (eg, antibiotics, anticoagulants, or opioids for pain), resulting in 27 discrete candidate low-value prescribing practices that were considered for inclusion in EVOLV-Rx (eTable 3 in the Supplement provides a full list of practices and definitions).

### Overall Ratings of the Expert Panel 

The round 1 ratings of the online modified-Delphi panel of experts for the scientific validity and clinical usefulness of each of the 27 candidate low-value prescribing practices are shown in Table 1. There was agreement among the panelists regarding the candidate practices except for prescribing statins for the primary prevention of atherosclerotic cardiovascular disease (ASCVD). At the conclusion of round 1, 19 candidate practices received a median score of 6.5 or higher, indicating high scientific validity. Eight candidate practices received a median score of 3.5 to 6 (an uncertain degree). Twenty candidate practices received a high clinical usefulness score of 6.5 or higher, and 7 candidate practices were rated as having uncertain clinical usefulness.

**Table 1.** Expert Panel Ratings and Characterizations of Scientific Validity and Clinical Usefulness of Candidate Low-Value Prescribing Practices

Candidate low-value prescribing practice	Median score^a^			
	Scientific validity		Clinical usefulness	
	Round 1	Round 3	Round 1	Round 3
Ineffective use^b^				
Thyroid hormone for subclinical hypothyroidism	8	8	7	8
Testosterone for nonspecific aging symptoms	8	8	8	8
Docusate sodium for constipation	7	7	8	8
Gabapentinoids for non-neuropathic pain	8	8	8	8
Prolonged use^c^				
PPIs	7	7	8	8
NSAIDs	8	8	7	7
DAPT after PCI	8	8	8	7
Inappropriate use^d^				
Vitamin B_12_ supplementation	7	7	7	7
Antipsychotic drugs in patients with dementia	8	8	7	7
Antibiotics for respiratory conditions	8	8	8	8
Antiparkinsonian medications in patients prescribed an antipsychotic drug or metoclopramide hydrochloride	7	7	7	7
AChE inhibitors for severe Alzheimer dementia	7	7	7	7
Potentially unsafe use^e^				
DAPT and systemic anticoagulation drugs	7.5	7.5	8	8
Benzodiazepines	8	8	7	8
Skeletal muscle relaxants	7	7	7	7
Anticholinergic drugs	7	7	7	7
Overly intensive treatment^f^				
Type 2 diabetes	8	8	8	8
COPD	8	8	8	8
Candidate prescribing practices that did not meet inclusion criteria				
Candidate prescribing practices rated as scientifically valid but of uncertain clinical usefulness				
Aspirin for primary prevention of ASCVD	6	7	6	6
Sedative or hypnotic sleeping aids	7	7	6	6
Opioids for treatment of noncancer pain	6	6.5	5	6
Candidate prescribing practices rated as having uncertain scientific validity				
Statins for primary prevention of ASCVD	5	5	4^g^	5
Inappropriate use of iron supplementation	6	6	6	6
Nitrofurantoin for the treatment or prevention of UTI	5	6	5	5
Loop diuretics with a calcium channel blocker as part of a prescribing cascade	6	6	7	7
Genitourinary antispasmodic drugs in patients prescribed a cholinesterase inhibitor as part of a prescribing cascade	6	6	6	6
Overtreatment of hypertension	6	6	7	7

Abbreviations: AChE, acetylcholinesterase; ASCVD, atherosclerotic cardiovascular disease; COPD, chronic obstructive pulmonary disease; DAPT, dual antiplatelet therapy; NSAID, nonsteroidal anti-inflammatory drug; PCI, percutaneous coronary intervention; PPI, proton pump inhibitor; UTI, urinary tract infection.

^a^ Key for scientific validity or clinical usefulness scores on a 9-point Likert scale: 1 to 3 indicating low validity or usefulness; 3.5 to 6, uncertain validity or usefulness; and 6.5 to 9, high validity or usefulness.

^b^ Ineffective use: use for a common indication despite evidence of minimal to no benefit and possible harm or excessive cost.

^c^ Prolonged use: use beyond a certain time threshold when the harms or costs may outweigh the benefits.

^d^ Inappropriate use: use for inappropriate indications for which the harms or costs may outweigh the benefits.

^e^ Potentially unsafe use: use in situations in which the harms may outweigh the benefits.

^f^ Overly intensive treatment: treatment involving an excessive dose or number of medications that may result in harm or excessive cost.

^g^ Represents disagreement in round 1 as determined by the RAND/UCLA Appropriateness Method.

After considering the panelist scores, comments, and deliberations from rounds 1 and 2, we revised the sensitive and/or specific criteria that defined 19 candidate low-value prescribing practices (eTable 3 in the Supplement). Round 3 ratings are also shown in Table 1. In round 3, 18 candidate practices received high validity and clinical usefulness scores of 6.5 or higher and were included in EVOLV-Rx (Table 2).

**Table 2.** Final Components of EVOLV-Rx Codified by the Expert Panel

Final low-value prescribing practice	Criteria for defining low-value prescribing	
	Base (sensitive) criteria: patients broadly subject to potential low-value prescribing	Additional specific criteria: patients satisfying any 1 of value-based criteria for each individual practice
Ineffective use^a^		
Thyroid hormone for subclinical hypothyroidism	Use in patients with subclinical hypothyroidism and no history or active diagnosis of hypothyroidism	Age ≥80 y, or new prescription for thyroid hormone with a TSH <10 mIU/L Use of a brand-name thyroid hormone replacement
Testosterone for nonspecific aging symptoms	Use without a diagnosis of hypogonadism or panhypopituitarism^b^	History of VTE, ASCVD, or prostate cancer Use of a brand-name or transdermal preparation
Docusate for constipation	Any use	Concurrent use with other laxatives Use without a history of hemorrhoids
Gabapentinoids for non-neuropathic pain	Use without a diagnosis of postherpetic neuralgia or neuropathic pain (excluding patients with a history of epilepsy)	Risk factors for fall or fracture^c^ History of CKD and a daily dose >900 mg Therapeutic duplication or concurrent use with an antidepressant or other high-risk psychoactive medication^d^ Use of brand-name gabapentin or pregabalin
Prolonged use^a^		
PPIs	Use for >2 consecutive mo	No guideline-concordant indication for prolonged use (eg, erosive esophagitis, refractory GERD) No concurrent use of chronic NSAIDs or steroids Use of a brand-name PPI
NSAIDs	Use for >90 consecutive d, excluding patients with pericarditis or a rheumatologic condition	COX-1 selective NSAID in patients at an increased risk for a GIB and not prescribed a PPI^e^ COX-2 selective NSAID in patients with ASCVD Any NSAID in patients aged ≥75 y or with CKD
DAPT after PCI	Use of DAPT for >6 mo in patients after PCI for stable ischemic heart disease	DAPT for >12 mo DAPT use for >6 mo in patients at increased risk of bleeding associated with a history of PUD or GIB or concurrent use of an anticoagulant
Inappropriate use^a^		
Vitamin B_12_ supplementation	Use without an appropriate or active diagnosis (anemia, B_12_ deficiency, or gastric bypass surgery)	Administered intramuscularly or subcutaneously
Antipsychotic drugs in patients with dementia	Use for >90 consecutive d in patients with dementia without evidence of an underlying serious mental illness that would otherwise warrant use	Prolonged QT or risk factors for fall or fracture^c^ Therapeutic duplication or concurrent use with another high-risk psychoactive medication^d^ Use of a brand-name antipsychotic
Antibiotics for respiratory conditions	Use of antibiotics for conditions where antibiotics have been characterized as sometimes indicated (eg, acute or chronic pharyngitis) or never indicated (eg, asthma exacerbation)^f^	Use for conditions where antibiotics have been characterized as never indicated (eg, asthma exacerbation)^f^ Use of a brand-name antibiotic
Antiparkinsonian medications in patients prescribed an antipsychotic drug or metoclopramide	Concurrent use of an antiparkinsonian medication and an antipsychotic drug or metoclopramide (excluding patients with a history of serious mental illness that would otherwise warrant use)	New use of an antiparkinsonian medication within 6 mo after receiving a new prescription for an antipsychotic drug or metoclopramide
AChE inhibitors for severe Alzheimer dementia	Use of an AChE inhibitor to treat severe or end-stage Alzheimer dementia	Risk factors for fall or fracture^c^ Use of a brand name dementia medication
Potentially unsafe use^a^		
DAPT and systemic anticoagulation	Any concurrent use of 2 antiplatelet agents and an anticoagulant for >1 mo	History of PUD, upper GIB, or coagulopathy Use of a brand-name antiplatelet agent
Benzodiazepines	Use for >4 wk without a guideline-concordant indication (ie, seizure disorder, severe generalized anxiety disorder)^g^	Risk factors for fall or fracture^c^ Therapeutic duplication or concurrent use with another high-risk psychoactive medication^d^ Use of a brand-name benzodiazepine drug
Skeletal muscle relaxants	Use for >4 total wk^g^	Risk factors for fall or fracture^c^ Therapeutic duplication or concurrent use with another high-risk psychoactive medication^d^ Use of a brand-name skeletal muscle relaxant
Anticholinergic drugs	Concomitant use of ≥2 highly anticholinergic drugs or medication classes^h^	Risk factors for fall or fracture^c^ Therapeutic duplication or concurrent use with another high-risk psychoactive medication^d^ Use of a brand-name anticholinergic drug
Overly intensive treatment^a^		
Type 2 diabetes	Use of >2 diabetes medications with an A_1C_ level <7.0	Age ≥75 y, history of hypoglycemia, or risk factors for fall or fracture^c^ Use of a high-risk medication, including sulfonylureas, meglitinides, or thiazolidinediones
COPD	Use of inhaled corticosteroids in adults with an active diagnosis of mild to moderate COPD (Gold Class A, B)	≤1 COPD exacerbation in the previous 1 y

Abbreviations: AChE, acetylcholinesterase; ASCVD, atherosclerotic cardiovascular disease; CKD, chronic kidney disease; COPD, chronic obstructive pulmonary disease; COX, cyclooxygenase; DAPT, dual antiplatelet therapy; EVOLV-Rx, Evaluating Opportunities to Decrease Low-Value Prescribing; GERD, gastroesophageal reflux disease; GIB, gastrointestinal bleeding; NSAID, nonsteroidal anti-inflammatory drug; PCI, percutaneous coronary intervention; PPI, proton pump inhibitor; PUD, peptic ulcer disease; TSH, thyroid-stimulating hormone; VTE, venous thromboembolism.

^a^ Ineffective use: use for a common indication despite evidence of minimal to no benefit and possible harm or excessive cost. Prolonged use: use beyond a certain time threshold when the harms or costs may outweigh the benefits. Inappropriate use: use for inappropriate indications for which the harms or costs may outweigh the benefits. Potentially unsafe use: use in situations in which the harms may outweigh the benefits. Overly intensive treatment: treatment involving an excessive dose or number of medications that may result in harm or excessive cost.

^b^ Absence of an *International Statistical Classification of Diseases and Related Health Problems, Tenth Revision* claim for hypogonadism or panhypopituitarism, or absence of a total testosterone value <300 ng/dL (to convert to nanomoles per liter, multiply by 0.0347), if laboratory data were available, in the 365 days before the first prescription of the study year.

^c^ Included those aged 80 years or older, unless otherwise stated; history of cognitive disorder, previous falls or fractures, or frailty, per the claims-based algorithms in Green et al^27^ and Kim et al.^28^

^d^ Included the following medications classes: opioids, benzodiazepines, muscle relaxers, anticonvulsants, and sedatives.

^e^ Included patients with a history of PUD, other forms of upper GIB, coagulopathy, or concurrent use with other anticoagulants or NSAIDs.

^f^ Per the claims-based algorithm in Fleming-Dutra et al.^29^

^g^ Use did not need to be consecutive and applied to PRN or standing dose.

^h^ Included the following medications or classes: bladder or intestinal antispasmodic drugs, tricyclic antidepressants, first-generation antihistamines, doxepin hydrochloride, and mirtazapine.

Of those candidate low-value prescribing practices that were not incorporated in the final metric, 3 (aspirin for primary prevention of ASCVD, opioids for treatment of noncancer pain, and potentially unsafe use of sedative or hypnotic sleeping aids) were rated as having high scientific validity but uncertain clinical usefulness (eTable 3 in the Supplement). Six candidate practices received an uncertain scientific validity rating. One candidate low-value prescribing practice had a median validity score of 5, whereas the other 5 candidate practices received a median score of 6 (Table 1). For example, for the practice of prescribing statins for the primary prevention of ASCVD (final median score of 5 in scientific validity), the panelists commented that trials were ongoing to evaluate the effectiveness of statins for the primary prevention of ASCVD in older adults and that data from observational studies were insufficient to rate this candidate practice as being scientifically valid. In another example, the practice of prescribing potentially unsafe use of antihypertension medications received a final median score of 6 in scientific validity. The explanation for this rating was the presence of conflicting evidence of the reasonable blood pressure target in older adults and the anticipated challenges in operationalizing this practice given other indications for common blood pressure medications, such as β-blockers.

## Discussion

We convened an online modified-Delphi panel of experts to codify the components of EVOLV-Rx (Table 2). Of the 27 candidate low-value prescribing practices derived from 527 low-value prescribing recommendations, the panel identified the 18 most scientifically valid and clinically useful prescribing practices and related criteria for detecting low-value prescribing in the care of older adults. In addition, these practices and criteria reflect the perspectives of patients, caregivers, and practicing physicians. EVOLV-Rx contains a set of quality indicators that may be scaled and automated to detect low-value prescribing in large administrative or other clinical data sets for thousands of patients. We believe this metric would enable health systems, third-party payers, and policy makers to reduce low-value prescribing in ways that are both clinically sound and broadly acceptable.

EVOLV-Rx is distinct from other metrics in that it applies a value-based paradigm to consolidate the hundreds of existing low-value prescribing recommendations to define, prioritize, and systematically measure low-value prescribing practices in a way that can be operationalized in payer and provider data. Kerr et al^14^ recently applied a similar approach to identify high-priority recommendations for deintensifying care within the Veterans Health Administration. Rather than develop additional broad-based guidelines, we sought to specify operational definitions for the components of a metric that were intended for use in administrative claims and other health data. Thus, because of measurement concerns, we excluded candidate low-value prescribing practices, such as the overtreatment of hypertension, from the final version of EVOLV-Rx. With clearly defined sensitive and specific criteria for each low-value prescribing practice, EVOLV-Rx may serve as a useful adjunct to the Schwartz low-value care metric, which contains 31 distinct low-value tests and procedures but not medications and is currently used by the Medicare Payment Advisory Commission, Veterans Health Administration, and private insurers.^30,31^

Furthermore, EVOLV-Rx is unique in the way it integrates the perspectives of patients, caregivers, and practicing physicians about low-value prescribing and health care value. The panelists acknowledged these perspectives in their deliberations. Consequently, the individual components of EVOLV-Rx reflect stakeholder views on specific medications and their adverse effects; medical comorbidities that make older patients susceptible to low-value prescribing; and burdensome and costly medication preparations and methods of administration, such as the unnecessary subcutaneous or intramuscular injection of vitamin B_12_. By incorporating the views of diverse stakeholders in the development of EVOLV-Rx, we believe we have increased the likelihood that, when applied in clinical practice, EVOLV-Rx will decrease patients’ medication burden in a way that aligns with their values, is less likely to be perceived as rationing, and has face validity for practicing clinicians who are tasked with its use.^32^

The application of EVOLV-Rx may advance a variety of research and policy priorities. There is growing interest in deprescribing low-value medications among older adults, as evidenced by the emergence of federally funded Deprescribing Research Networks in the US and in other countries. EVOLV-Rx may help researchers, third-party payers, and health system leaders involved in deprescribing efforts to identify a research focus, given the hundreds of low-value prescribing recommendations, and conduct more rigorous audit and feedback of prescribing practices.^33,34^ From a policy perspective, Medicare has been increasingly discouraging the provision of low-value care through value-based payment reforms.^35^ In addition, third-party payers are implementing arrangements, such as bundled payments and shared savings plans, to promote the delivery of high-value care.^36^ The application of EVOLV-Rx may enable such efforts to readily incorporate low-value prescribing.

### Limitations

This study and EVOLV-Rx have several limitations. First, the low-value prescribing practices contained in the metric are not exhaustive given that our goal was to create a focused, acceptable, and scalable metric. Therefore, its use does not preclude the application of traditional tools, such as the Beers Criteria. Second, the infographics we provided to each panelist were intended to guide their deliberations but did not depict the findings of a systematic review for each candidate low-value prescribing practice. However, each infographic underwent peer review to ensure its accuracy and each included a summary, links, and references to systematic reviews or guidelines based on such reviews. The panelists were instructed to use these infographics as a reference or supplementary material rather than as a sole resource for their deliberations and when rating each candidate low-value prescribing practice. Third, the results (the criteria in EVOLV-Rx) are sensitive to the composition of the Delphi panel, and it is not known to what degree these results would be different with a different set of experts. Fourth, EVOLV-Rx has not yet been fully operationalized for use in administrative claims or electronic health record data. The research team is currently working to operationalize and establish the specification validity of EVOLV-Rx. Fifth, each component of EVOLV-Rx may not be applicable in all forms of health data (eg, administrative claims vs electronic health records) or in all populations, which was a necessary tradeoff in developing criteria that best characterize each form of low-value prescribing.

## Conclusions

In this qualitative study, a panel of experts identified 18 scientifically valid and clinically useful prescribing practices and related criteria for detecting low-value prescribing practices in the care of older adults, resulting in the development of the EVOLV-Rx metric. The application of EVOLV-Rx may also enhance the detection of low-value prescribing alongside other low-value tests and procedures, reduce polypharmacy, and enable older adults to receive high-value care across the full spectrum of health services in a way that aligns with their perspectives and values.

## Notes

Supplement. **eTable 1.** Low-Value Prescribing Practice Candidates Solicited from Patients, Caregivers, and Practicing Physicians **eFigure.** Infographic Examples for 3 Candidate LVP Metrics **eTable 2.** Questions Posed to the Delphi Panelists to Assess Scientific Validity and Usefulness in Clinical Practice for Each Candidate LVP Metric **eTable 3.** Changes Made to the Sensitive and Specific Criteria for Each Candidate LVP Metric After Rounds 1 and 2
