# Supplementary material for: Development of a Metric to Detect and Decrease Low-Value Prescribing in Older Adults
Source: JAMA Netw Open. 2022 Feb 15;5(2):e2148599. doi: 10.1001/jamanetworkopen.2021.48599 (PMC8848205; doi:10.1001/jamanetworkopen.2021.48599)
Supplement: Supplement. — eTable 1. Low-Value Prescribing Practice Candidates Solicited from Patients, Caregivers, and Practicing Physicians eFigure. Infographic Examples for 3 Candidate LVP Metrics eTable 2. Questions Posed to the Delphi Panelists to Assess Scientific Validity and Usefulness in Clinical Practice for Each Candidate LVP Metric eTable 3. Changes Made to the Sensitive and Specific Criteria for Each Candidate LVP Metric After Rounds 1 and 2 [file jamanetwopen-e2148599-s001.pdf]

Supplementary Online Content

Radomski TR, Decker A, Khodyakov D, et al. Development of a metric to detect and decrease low-value prescribing in older adults. *JAMA Netw Open*. 2022;5(2):e2148599. doi:10.1001/jamanetworkopen.2021.48599

eTable 1. Low-Value Prescribing Practice Candidates Solicited from Patients, Caregivers, and Practicing Physicians

eFigure. Infographic Examples for 3 Candidate LVP Metrics

eTable 2. Questions Posed to the Delphi Panelists to Assess Scientific Validity and Usefulness in Clinical Practice for Each Candidate LVP Metric

eTable 3. Changes Made to the Sensitive and Specific Criteria for Each Candidate LVP Metric After Rounds 1 and 2

This supplementary material has been provided by the authors to give readers additional information about their work.

© 2022 Radomski TR et al. *JAMA Network Open.*

eTable 1. Low-Value Prescribing Practice Candidates Solicited from Patients, Caregivers, and Practicing Physicians

Patients	Caregivers	Practicing Physicians
**Specific Medications/Classes:**<br>Insulin<br>Metformin<br>Liraglutide<br>Aspirin<br>Metoprolol<br>Diltiazem<br>Hydrochlorothiazide<br>Lisinopril<br>Antihypertensive, did not specify type<br>Diuretic, did not specify type<br>Atorvastatin<br>Pravastatin<br>Rosuvastatin<br>Statin, did not specify type<br>Warfarin<br>Ticagrelor<br>Clopidogrel<br>Ranolazine<br>Albuterol inhaler<br>Inhaler, did not specify type<br>Flonase<br>Fluoxetine<br>Gabapentin<br>Carbamazepine<br>Phenytoin<br>Medication for bipolar disorder, did not specify<br>MS medication, did not specify<br>Amantadine<br>Meloxicam<br>NSAIDs<br>Acetaminophen<br>Oxycodone<br>Morphine<br>Codeine<br>Antibiotic, did not specify type<br>Prednisone<br>Calcium supplement<br>Fosamax<br>Fish oil<br>Vitamins, did not specify type<br>Probiotic<br>Omeprazole<br>Eye drops for cataract, did not specify type<br>Restasis<br>HCV medication, did not specify type	**Specific Medications/Classes:**<br>Insulin<br>Metformin<br>Furosemide<br>Antihypertensive, did not specify type<br>SL nitroglycerin<br>Atorvastatin<br>Albuterol inhaler<br>Inhaler, did not specify type<br>Bupropion<br>Sertraline<br>Lorazepam<br>Melatonin<br>Antidepressant, did not specify type<br>Anti-anxiety medication, did not specify type<br>Morphine<br>Oxycodone<br>Naloxone<br>Rivastigmine<br>Antibiotic, did not specify type<br>Oral chemotherapy, did not specify type<br>Eye drops (for glaucoma), did not specify type<br>Lidocaine cream<br>Laxatives, did not specify type<br>Suppository, did not specify type<br>Vitamins, did not specify type<br><br>**Medication Characteristics (applies to patients and caregivers):**<br>Medications with common side effects that may impact quality of life, including but not limited to:<br>- drowsiness/fatigue<br>- dizziness/lightheadedness<br>- gastrointestinal upset<br>- muscle cramps<br>- rash<br>- confusion/altered mental status<br><br>Medications that are inconvenient to administer:<br>- inconvenient times<br>- need to take with food<br>- associated laboratory or point of care testing<br><br>Medications that are uncomfortable to administer:<br>- large pills (difficult to swallow<br>- injectable medications<br><br>Any medications whose costs required patients to make material sacrifices in other areas of their life.	**Specific Medications/Classes:**<br>Aspirin<br>Statins<br>Proton Pump Inhibitors<br>Stool Softeners<br>Beta Blockers<br>Vitamin D<br>Calcium<br>ACE Inhibitors<br>Vitamins (multivitamin/iron/B12/E)<br>Donepezil<br>Topical analgesics/lidocaine patch<br>Antihistamines<br>H2 Blockers<br>Opiates<br>Cough suppressants<br>Insulin<br>Non-generics<br>Anticoagulants<br>Alendronate<br>Weight loss/energy<br>Oxybutynin<br>Insomnia medication<br>Benzodiazepines<br>Antiepileptics<br>NSAIDs<br>Memantine<br>Mirtazapine<br>Antihypertensives<br>Gout prevention<br>Anti-oxidants<br>Inhalers<br><br>**Medication Characteristics:**<br>Potential for adverse drug events including but not limited<br>- bleeding<br>- falls<br>- acute kidney injury<br><br>Medications that require a prolonged period to have an effect (e.g., statins)<br><br>Medications that may interact with patients' underlying disease states including but not limited to the following:<br>- Chronic kidney disease<br>- frailty<br>- multimorbidity<br>- gait distburbances<br><br>Other patient-level considerations:<br>- inability to pay<br>- inability to administer complex regimen

[a] Low-value medications expressly mentioned by the study participants, as well as medications whose characteristics were consistent with a low-value prescribing feature cited by study participants (e.g., inconvenient route or timing of administration, side effect profile), were considered for inclusion

eFigure. Infographic Examples for 3 Candidate LVP Metrics

ASPIRIN FOR PRIMARY PREVENTION OF ATHEROSCLEROTIC CARDIOVASCULAR DISEASE

PROPOSED LOW-VALUE PRESCRIBING PRACTICE DEFINITION

Baseline (Sensitive) Criterion

Use of a baby aspirin (81mg) in adults aged ≥70 without a diagnosis of atherosclerotic cardiovascular disease (ASCVD)*

*ASCVD includes coronary artery disease, cerebrovascular disease, and peripheral arterial disease

Additional Specific Criteria

(Any one of the following)

- History of peptic ulcer disease, other form of upper gastrointestinal bleeding, or coagulopathy (H)
- Concurrent use with other anticoagulants or other NSAIDs (H)

SYNOPSIS OF THE EVIDENCE

- Daily aspirin use among older adults to reduce the risk of heart attack and stroke is a long-standing practice, affecting nearly 20 million adults. However, recent trials have questioned the efficacy and safety of daily aspirin prescribed for this indication (1).
- The ASPREE randomized clinical trials demonstrated that among adults aged 70 years or older, taking enteric-coated aspirin for the primary prevention of cardiovascular disease did not prolong disability-free survival or lower the risk of developing cardiovascular disease, but did result in a significantly higher risk of major hemorrhage (2, 3, 4).
- The ASCEND trial demonstrated that among patients with diabetes, taking aspirin prevented serious cardiovascular events, but that these events were counter-balanced by major bleeding events (5).
- In a systematic review and meta-analysis of 13 randomized clinical trials, the use of aspirin for primary prevention of cardiovascular disease was associated with a lower risk of cardiovascular events but an increased risk of major bleeding (6).

19,700,000 The number of adults who took aspirin for primary prevention in 2017 (1)

© 2022 Radomski TR et al. *JAMA Network Open.*

CURRENT GUIDELINES

RECOMMENDATIONS CAUTIONING AGAINST USE

MEDICATION LIST	SUMMARY OF RECOMMENDATIONS
Choosing Wisely	—
Beers List	—
STOPP/START	Stop long-term aspirin at doses greater than 150mg/day.<br>Stop aspirin use with concurrent significant bleeding risk.
FORTA	—

RECOMMENDATIONS FOR APPROPRIATE USE*

PROFESSIONAL ORGANIZATION	AGE GROUPS 50-59	60-69	≥70
ACCP (2012)	✓	✓	✓
ADA (2016)	+/-	+/-	+/-
USPSTF (2016)	✓	+/-	✗
AHA/ACC (2019)	+/-	+/-	✗

*Recommendations apply to a patient with an ASCVD risk >10%

Abbreviations: ACCP, American College of Chest Physicians; ADA, American Diabetes Association; USPSTF, United States Preventive Services Task Force; AHA, American Heart Association; ACC, American College of Cardiology

✓ Use recommended
+/- Weigh risks and benefits
✗ Do not prescribe

STAKEHOLDER PERSPECTIVES

PATIENTS AND CAREGIVERS

✓
- Preventive medications valued less than medications used to treat symptoms

PRACTICING PRESCRIBERS

✓
- Recognition that aspirin is ineffective
- Prescribing should be individualized

*Pickering AN, Radomski TR, et al. Older Patient and Caregiver Perspectives on Medication Value and Deprescribing: A Qualitative Study. Journal of the American Geriatric Society. 2020; 68(4): 746-753

REFERENCES

1 O'Brien CW, Juraschek SP, Wee CC. Prevalence of Aspirin Use for Primary Prevention of Cardiovascular Disease in the United States: Results From the 2017 National Health Interview Study. Annals of Internal Medicine. 2019;171(8):596-598. PMID: 31330542

2 McNeil JJ, Woods RL, Nelson MR, et al. Effect of Aspirin on Disability-free Survival in the Healthy Elderly. New England Journal of Medicine. 2018;379(16):1499-1508. PMID: 30221596

3 McNeil JJ, Nelson MR, Woods RL, et al. Effect of Aspirin on All-Cause Mortality in the Healthy Elderly. New England Journal of Medicine. 2018;379(16):1519-1528. PMID: 30221595

4 McNeil JJ, Wolfe R, Woods RL, et al. Effect of Aspirin on Cardiovascular Events and Bleeding in the Healthy Elderly. New England Journal of Medicine. 2018;379(16):1509-1518. PMID: 30221597

5 Bowman L, Mafham M, Wallendszus K, et al. Effects of Aspirin for Primary Prevention in Persons with Diabetes Mellitus. New England Journal of Medicine. 2018;379(16):1529-1539. PMID: 30146931

6 Zheng SL, Roddick AJ. Association of Aspirin Use for Primary Prevention With Cardiovascular Events and Bleeding Events: A Systematic Review and Meta-analysis. JAMA. 2019;321(3):277-287. PMID: 30667501

This infographic was developed by Dr. Thomas Radomski as part of an NIH K23 Award. Please contact Dr. Radomski (Study PI - radomskitr@upmc.edu) to request permission if you wish to use or disseminate this infographic outside of the study.

© 2022 Radomski TR et al. *JAMA Network Open.*

UNNECESSARY USE OF ANTIBIOTICS FOR RESPIRATORY CONDITIONS

PROPOSED LOW-VALUE PRESCRIBING PRACTICE DEFINITIONS

Baseline (Sensitive) Criterion

Use of antibiotics for conditions where antibiotics have been characterized as "sometimes" (e.g. acute or chronic pharyngitis) or "never" indicated" (e.g. asthma exacerbation)*

*Antibiotic appropriateness classification: Fleming-Dutra KE, et al. JAMA. 2016; 315(17): 1864-1873.

Additional Specific Criteria

(Any one of the following)

- Use for conditions where antibiotics have been characterized as "never indicated" (e.g. asthma exacerbation) (E)*
- Use of a brand-name antibiotic (C)

SYNOPSIS OF THE EVIDENCE

- Antibiotics are among the most commonly inappropriately prescribed medications to Medicare beneficiaries, resulting in antibiotic resistance, increased costs of care, and other complications such as Clostridium difficile diarrhea. In 2010 and 2011, 12.6% of office visits resulted in an antibiotic prescription. Collectively, visits for acute respiratory conditions led to 221 antibiotic prescriptions per 1000 individuals (95% CI, 198-245) annually, of which only half were estimated to be appropriate (1-3).
- From 2011 – 2014, potentially inappropriate antibiotic claims among Medicare beneficiaries fell slightly from 553 to 522 per 1000 beneficiaries, with a notable decrease in the claims/beneficiary of azithromycin (-18.5%) and an increase in the use of levofloxacin (+27.7%) for respiratory conditions (1). However, among all adults in 2016, more than two-thirds of antibiotics and 91% of non-azithromycin courses prescribed for acute sinusitis were 10 days or longer, in contrast to IDSA guidelines that such courses of treatment be 5-7 days in duration (4).
- In a cohort study by Stefan et al, propensity score-matched analyses demonstrated that treatment with antibiotics in patients experiencing an asthma exacerbation resulted in 29% longer hospital stays (length of stay ratio, 1.29; 95% CI, 1.27-1.31) and higher cost of hospitalization (median [IQR] cost, $4776 [$3219-$7373] vs $3641 [$2346-$5942]) but with no difference in the risk of treatment failure (OR, 0.95; 95% CI, 0.82-1.11) (5).
- In a qualitative study , physicians reported that non-clinical factors frequently influenced their decisions to prescribe antibiotics and weighed the potential for side effects against patient satisfaction when making a decision (6).

USE AND COST IN MEDICARE PART D (2017)

- **27,137,284** Total beneficiaries
- **$657,192,710** Total spending
- **$1,045 vs $16** Average annual spending per beneficiary (Brand vs Generic)

*Values represent use of antibiotics in the top 50 medications prescribed to Medicare beneficiaries.

© 2022 Radomski TR et al. *JAMA Network Open.*

CURRENT GUIDELINES

RECOMMENDATIONS CAUTIONING AGAINST USE

MEDICATION LIST	SUMMARY OF RECOMMENDATIONS
Choosing Wisely	–
Beers List	–
STOPPFrail	–
FORTA	For Chronic Obstructive Pulmonary Disease (COPD) exacerbations, use antibiotics in cases of exacerbation, after calculated selection and, if necessary, according to antibiogram.

RECOMMENDATIONS FOR APPROPRIATE USE*

PROFESSIONAL ORGANIZATION	RECOMMENDATION
IDSA (2012)	Antibiotics should be only be prescribed for acute rhinosinusitis in the setting of symptoms lasting >10 days, worsening or new symptoms after day 5, or the onset of severe symptoms and fevers >39C lasting 3-4 days at the beginning of the illness.
GIA (2019)	Do not routinely prescribe antibiotics for asthma exacerbations.

Abbreviations: IDSA, Infectious Disease Society of America; GIA : Global Initiative for Asthma

STAKEHOLDER PERSPECTIVES

PATIENTS AND CAREGIVERS

- Don't take unless necessary due to potential adverse consequences (C. diff diarrhea)

PRACTICING PRESCRIBERS

- Use of antibiotics for upper respiratory infections frequently characterized as inappropriate

*Pickering AN, Radomski TR, et al. Older Patient and Caregiver Perspectives on Medication Value and Deprescribing: A Qualitative Study. Journal of the American Geriatric Society. 2020; 68(4): 746-753

REFERENCES

1 Olesen SW, Barnett ML, MacFadden DR, et al. Trends in outpatient antibiotic use and prescribing practice among US older adults, 2011-15: observational study. BMJ. 2018;362:k3155. Published 2018 Jul 27. doi:10.1136/bmj.k3155

2 Fleming-Dutra KE, Hersh AL, Shapiro DJ, et al. Prevalence of Inappropriate Antibiotic Prescriptions Among US Ambulatory Care Visits, 2010-2011. JAMA. 2016;315(17):1864–1873. doi:10.1001/jama.2016.4151

3 Tobia CC, Aspinall SL, Good CB, et al. Appropriateness of antibiotic prescribing in veterans with community-acquired pneumonia, sinusitis, or acute exacerbations of chronic bronchitis: a cross-sectional study. Clin Ther. 2008;30(6):1135-1144. doi:10.1016/j.clinthera.2008.06.009

4 King LM, Sanchez GV, Bartoces M, et al. Antibiotic Therapy Duration in US Adults With Sinusitis. JAMA Intern Med. 2018;178(7):992-994. doi:10.1001/jamainternmed.2018.0407

5 Stefan MS, Shieh M, Spitzer KA, et al. Association of Antibiotic Treatment With Outcomes in Patients Hospitalized for an Asthma Exacerbation Treated With Systemic Corticosteroids. JAMA Intern Med. 2019;179(3):333–339. doi:10.1001/jamainternmed.2018.5394

6 Patel A, Pfoh ER, Misra Hebert AD, et al. Attitudes of High Versus Low Antibiotic Prescribers in the Management of Upper Respiratory Tract Infections: a Mixed Methods Study. J Gen Intern Med. 2020;35(4):1182-1188. doi:10.1007/s11606-019-05433-5

This infographic was developed by Dr. Thomas Radomski as part of an NIH K23 Award. Please contact Dr. Radomski (Study PI - radomskitr@upmc.edu) to request permission if you wish to use or disseminate this infographic outside of the study.

© 2022 Radomski TR et al. *JAMA Network Open.*

PROLONGED USE OF PROTON PUMP INHIBITORS

PROPOSED LOW-VALUE PRESCRIBING PRACTICE DEFINITIONS

Base (Sensitive) Criterion

Use for greater than 2 consecutive months

Additional Specific Criteria
(Any one of the following)

- No guideline concordant indication for prolonged use (e.g. erosive esophagitis, refractory GERD, etc*) (E)
- No concurrent use of chronic NSAIDs or steroids (E)
- Use of a brand-name PPI (C)

*Link to guideline concordant indications

SYNOPSIS OF THE EVIDENCE

- Proton pump inhibitor (PPI) therapy is common among older adults, affecting greater than 10 million Medicare Part D beneficiaries. In a single center study, 1/3 of PPI prescriptions in older adults were potentially low-value (1).
- In several observational studies, older adults have been shown to be at increased risk of experiencing adverse consequences related to PPI use, including but not limited to dementia, chronic kidney disease, and hip fractures:
  - In a prospective cohort study of ~75,000 older adults, PPI use was associated with a 44% increased risk of dementia (HR 1.44, 95% CI 1.36-1.52) (2).
  - In a retrospective cohort study of 10,482 adults with a mean age of 63, PPI use was associated with an increased risk of incident Chronic Kidney Disease (HR 1.50, 95% CI 1.14-1.96) (3).
  - In a systematic review and meta-analysis of observational studies, PPI use was associated with an increased risk of hip fracture (OR 1.42, 95% CI 1.33-1.53)(4).
- In a randomized controlled trial of adults (mean age 68 years) prescribed pantoprazole, use up to 3 years was not associated with adverse events , except increased risk of enteric infections (5).

COST AND USE IN MEDICARE PART D (2017)*

- **12,122,05** Total beneficiaries
- **$2,384,836,248** Total spending
- **$1922 vs $106** Average annual spending per beneficiary (brand vs generic)

*Values represent prescription use for any reason

© 2022 Radomski TR et al. *JAMA Network Open.*

CURRENT GUIDELINES

RECOMMENDATIONS CAUTIONING AGAINST USE

MEDICATION LIST	SUMMARY OF RECCOMMENDATIONS
Choosing Wisely	Long-term acid suppression therapy should be titrated to the lowest effective dose needed to achieve therapeutic goals.
Beers List	Avoid scheduled use for >8 weeks unless for high-risk patients, erosive and Barrett esophagitis, pathological hypersecretory condition, or demonstrated need for maintenance treatment.
STOPPFrail	Avoid PPIs at full therapeutic dose, unless persistent dyspeptic symptoms at lower dose.
FORTA	Use PPIs only if necessary. If older patient must be treated with NSAID, then concurrent PPI should be standard of care.

RECOMMENDATIONS FOR APPROPRIATE USE*

PROFESSIONAL ORGANIZATION	Duration of Therapy: 4-8 weeks	Duration of Therapy: >8 weeks
CFPC (2017)	Uncomplicated GERD, mild to moderate esophagitis, Upper GI symptoms with resolution for 3 days	Continue or consult with gastroenterologist if history of Barrett's esophagus, chronic NSAIDS, severe esophagitis, or history of bleeding ulcer
AGA (2017)	Uncomplciated GERD	Continue if Barrett's esophagus, symptomatic GERD, or chronic NSAID use

*Abbreviation: CFPC, College of Family Physicians of Canada; AGA, American Gastroenterological Society

STAKEHOLDER PERSPECTIVES

PATIENTS AND CAREGIVERS

- Valued by patients as a medication that improves symptoms

PRACTICING PRESCRIBERS

- Continued unnecessarily beyond appropriate duration

*Pickering AN, Radomski TR, et al. Older Patient and Caregiver Perspectives on Medication Value and Deprescribing: A Qualitative Study. Journal of the American Geriatric Society. 2020; 68(4): 746-753

REFERENCES

1 Mafi JN, May FP, Kahn KL. Low-Value Proton Pump Inhibitor Prescriptions Among Older Adults at a Large Academic Health System. J Am Geriatr Soc. 2019;67(12):2600-2604. PMID: 31486549

2 Gomm W, Von Holt K, Thome F, et al. Association of Proton Pump Inhibitors With Risk of Dementia A Pharmacoepidemiological Claims Data Analysis. JAMA Neurol. 2016;73(4):410-416. doi:10.1001/jamaneurol.2015.4791

3 Lazarus B, Chen Y, Wilson FP, et al. Proton Pump Inhibitor Use and the Risk of Chronic Kidney Disease. JAMA Intern Med. 2016.176(2):238-246. doi:10.1001/jamainternmed.2015.7193

4 Islam M, Poly TN, Walther BA, et al. Adverse outcomes of long-term use of proton pump inhibitors: a systematic review and meta-analysis. European Journal of Gastroenterology & Hepatology. 2018.30(12):1395-1405. doi:doi.org/10.1097/MEG.0000000000001198

5 Moayyedi P, Eikelboom JW, Bosch J, et al. Safety of Proton Pump Inhibitors Based on a Large, Multi-Year, Randomized Trial of Patients Receiving Rivaroxabanor Aspirin. Gastroenterology. 2019.157(3):682-691. doi:https://doi.org/10/1053/j.gastro.2019.05.056

This infographic was developed by Dr. Thomas Radomski as part of an NIH K23 Award. Please contact Dr. Radomski (Study PI - radomskitr@upmc.edu) to request permission if you wish to use or disseminate this infographic outside of the study.

© 2022 Radomski TR et al. *JAMA Network Open.*

eTable 2. Questions Posed to the Delphi Panelists to Assess Scientific Validity and Usefulness in Clinical Practice for Each Candidate LVP Metric

When rating the **scientific validity** of each candidate low-value prescribing (LVP) metric, please consider whether there is adequate scientific evidence to suggest that the potential harms or costs associated with the candidate LVP metric typically outweigh the potential benefits. We want you to interpret the rating scores as follows:<br><br>• Scores of 1-to-3 indicate that this candidate LVP metric has low validity, meaning that there is insufficient evidence to warrant its inclusion.<br>• Scores of 4-to-6 indicate that this candidate LVP metric has uncertain validity, meaning that there is a moderate amount or conflicting evidence that warrant its inclusion.<br>• Scores of 7-to-9 indicate that this candidate LVP metric has high validity, meaning that there is strong evidence to warrant its inclusion.
When rating **usefulness in clinical practice**, please consider the sum total of your professional experiences to indicate whether you would use or recommend the use of this metric in clinical practice to improve the value of prescribing among older adults. When answering this question, please be sure to describe any considerations that influence your rating, especially if you feel the metric is otherwise sufficiently valid to warrant inclusion. We want you to interpret the rating scores as follows:<br><br>• Scores of 1-to-3 indicate that this LVP metric is of low usefulness, meaning that you would be unlikely to use or recommend the use of this metric in clinical practice to improve the value of prescribing among older adults.<br>• Scores of 4-to-6 indicate that this LVP metric is of uncertain usefulness, meaning that you are unsure of or ambivalent about recommending the use of this metric in clinical practice to improve the value of prescribing among older adults.<br>• Scores of 7-to-9 indicate that this LVP metric is of high usefulness, meaning that you would be likely to use or recommend the use of this metric in clinical practice to improve the value of prescribing among older adults.

© 2022 Radomski TR et al. *JAMA Network Open.*

eTable 3. Changes Made to the Sensitive and Specific Criteria for Each Candidate LVP Metric After Rounds 1 and 2

**Individual Low-Value Prescribing Practices**	**Low-Value Prescribing Criteria[a]**	
	**Base (Sensitive) Criteria**<br>Captures patients broadly subject to potential low-value prescribing	**Additional Specific Criteria**<br>Patients flag for inclusion by satisfying any one of the following value-based criteria for each individual metric
**Ineffective Use** (Use for a common indication despite evidence of minimal to no benefit and possible harm)		
Thyroid hormone for sub-clinical hypothyroidism	Use in patients with sub-clinical hypothyroidism and no history or active diagnosis of hypothyroidism	Age ≥80<br><br>New prescription for thyroid replacement hormone with a TSH <10 mIU/L<br><br>Use of a brand-name thyroid hormone replacement
Testosterone for non-specific aging symptoms	Use without a diagnosis of hypogonadism or pan-hypopituitarism<br><br>**(Absence of an ICD claim for hypogonadism or pan-hypopituitarism, or the absence of a total testosterone value <300 ng/dL (if lab data available) in the 365 days prior to the first prescription of the study year)**	History of venous thromboembolism, atherosclerotic cardiovascular disease, or prostate cancer<br><br>Use of a brand-name or transdermal preparation
Docusate for constipation	Any use	Concurrent use with other laxatives<br><br>Use without a history of hemorrhoids
Gabapentinoids for non-neuropathic pain	Use without a diagnosis of post-herpetic neuralgia or neuropathic pain<br><br>(excluding patients with a history of epilepsy)	Risk factors for fall/fracture[b]<br><br>**History of CKD and a daily dosage greater than 900mg**<br><br>Therapeutic duplication or concurrent use with an opioid, benzodiazepine, muscle relaxer, anti-depressant, anticonvulsant, or sedative sleeping medication<br><br>Use of brand-name gabapentin or pregabalin

© 2022 Radomski TR et al. *JAMA Network Open.*

**Prolonged Use** (Use beyond a certain time threshold where the harms or costs may outweigh the benefits)		
Proton pump inhibitors	Use for greater than 2 consecutive months	No guideline concordant indication for prolonged use (e.g., erosive esophagitis, refractory GERD, etc.)<br><br>No concurrent use of chronic NSAIDs or steroids<br><br>Use of a brand-name PPI
Non-steroidal anti-inflammatories	Use **>90** consecutive days, **excluding patients with pericarditis or a rheumatologic condition**<br><br>(Use applies to ~~PRN or~~ standing dosing)	COX-1 selective NSAID in patients at an increased risk of experiencing a GI bleed and not prescribed a PPI[c]<br><br>COX-2 selective NSAID in patients with atherosclerotic cardiovascular disease<br><br>Any NSAID (COX 1 or 2) in pts **Aged ≥75** OR chronic kidney disease
Dual-anti platelet therapy after PCI	Use of dual anti-platelet therapy (DAPT) for >6 months in patients who underwent PCI for stable ischemic heart disease	DAPT >12 months<br><br>DAPT use > 6 months in patients at increased risk of bleeding due to a history of peptic ulcer disease/gastrointestinal bleeding, or concurrent use of an anticoagulant
**Inappropriate Use** (Medications commonly prescribed for inappropriate indications)		
Vitamin B12 supplementation	Use without an appropriate or active diagnosis (e.g., anemia, vitamin B12 deficiency, or history of gastric bypass surgery)<br><br>**(Vitamin B12 deficiency defined as an ICD code for Vitamin B12 deficiency OR B12 value <350pg/mL in the 365 days prior to the first prescription of the study year)**	AND administered intramuscularly or subcutaneously

© 2022 Radomski TR et al. *JAMA Network Open.*

Anti-psychotics in patients with dementia	Use **for >90 consecutive days** in patients with dementia without evidence of an underlying serious mental illness that would otherwise warrant use	Prolonged QT or Risk factors for fall/fracture[b]<br><br>Therapeutic duplication or concurrent use with opioids, muscle relaxants, anti-convulsants, or sedative sleeping medications<br><br>Use of a brand-name antipsychotic
Antibiotics for respiratory conditions	Use of antibiotics for conditions where antibiotics have been characterized as "sometimes" (e.g., acute or chronic pharyngitis) or "never" indicated (e.g., asthma exacerbation)~~, or if indication is unknown~~[d]	Use for conditions where antibiotics have been characterized as "never" indicated (e.g., asthma exacerbation)[d]<br><br>Use of a brand-name antibiotic
Anti-parkinsonian medications in patients prescribed an anti-psychotic or metoclopramide	Concurrent use of an anti-parkinsonian medication and an anti-psychotic medication or metoclopramide<br><br>**(Excluding patients with a history of serious mental illness that would otherwise warrant use)**	New use of an anti-parkinsonian medication within 6 months after receiving a new prescription for an anti-psychotic medication or metoclopramide<br><br>~~Use of a brand name anti-parkinsonian medication~~
AchE inhibitors for Severe Alzheimer's Dementia	Use of an **acetylcholinesterase inhibitor** to treat severe or end stage Alzheimer's Dementia<br>~~without an appropriate indication (e.g., use in patients with mild cognitive impairment, severe, or end stage dementia)~~	Risk factors for fall/fracture[b]<br><br>Use of a brand name dementia medication
**Potentially Unsafe Use** (Use where the harms are likely to outweigh the benefits)		
Dual anti-platelet therapy and systemic anticoagulation	Any concurrent use of two anti-platelet agents and an anticoagulant for > 1 month	History of peptic ulcer disease, other form of upper gastrointestinal bleeding, or coagulopathy<br><br>Use of a brand name anti-platelet medication

© 2022 Radomski TR et al. *JAMA Network Open.*

Benzodiazepines	Use for >4 weeks without a guideline concordant indication (i.e., seizure disorder, severe generalized anxiety disorder)<br><br>(Use does not need to be consecutive and applies to PRN or standing dosing)	Risk factors for fall/fracture[b]<br><br>Therapeutic duplication or concurrent use with an opioid, muscle relaxer, anti-convulsant, or sedative sleeping medication<br><br>Use of a brand-name benzodiazepine
Skeletal muscle relaxants	~~Any use of skeletal muscle relaxants in older adults~~<br><br>**Use for >4 total weeks**<br><br>**(Use does not need to be consecutive and applies to PRN or standing dosing)**	Risk factors for fall/fracture[b]<br><br>Therapeutic duplication or concurrent use with an opioid, benzodiazepine, anti-convulsant, or sedative sleeping medication<br><br>Use of a brand name skeletal muscle relaxant
Anti-cholinergic medications	~~Any use of an anti-cholinergic medication rated as a 3 on the anticholinergic cognitive burden score~~<br><br>**Concomitant use of 2 or more highly anticholinergic medications or medication classes**<br><br>**(includes the following medications/classes: bladder or intestinal antispasmodics, tricyclic antidepressants, first generate anti-histamines, doxepin, and mirtazapine)**	Risk factors for fall/fracture[b]<br><br>Therapeutic duplication or concurrent use with an opioid, benzodiazepine, muscle relaxer, anti-convulsant, or sedative sleeping medication<br><br>Use of a brand name anticholinergic agent
**Overly Intensive Treatment**		

© 2022 Radomski TR et al. *JAMA Network Open.*

Type 2 diabetes mellitus	Use of **>2** diabetes medications with an A1C <7.0	Age ≥75, history of hypoglycemia, or risk factors for fall/fracture[b]<br><br>Use of a high risk medication, including sulfonylureas, meglitinides, or thiazolidinediones
Chronic Obstructive Pulmonary Disease	Use of inhaled corticosteroids in adults with an active diagnosis of mild to moderate COPD (Gold Class A, B)	AND ≤1 COPD exacerbation in the prior 1 year
**Candidate Prescribing Practices Not Included in the Final Metric**		
**Candidate Prescribing Practices Rated as Scientifically Valid but of Uncertain Clinical Usefulness**		
Aspirin for primary prevention of ASCVD	Use of a baby aspirin (81mg) in adults **aged ≥70** without a diagnosis of atherosclerotic cardiovascular disease (ASCVD)<br><br>**(ASCVD includes coronary artery disease, cerebrovascular disease, and peripheral arterial disease)**	History of peptic ulcer disease, other form of upper gastrointestinal bleeding, or coagulopathy<br><br>Concurrent use with other anticoagulants or other NSAIDs<br><br>~~<10%10-year ASCVD risk~~
Opioids for treatment of non-cancer pain	**Use of an opioid prescription with an average daily dosage of ≥90 morphine milligram equivalents (MME) over a period of ≥90 days**<br><br>(Excludes use of buprenorphine, or treatment for cancer-related or **sickle cell related pain)**	Overdose or risk factors for fall/fracture[b]<br><br>Therapeutic duplication or concurrent use with a benzodiazepine, muscle relaxer, anti-convulsant, or sedative sleeping medication<br><br>Use of a brand-name opioid
Sedative/hypnotic sleeping aids	Use for >4 total weeks (does not need to be consecutive)<br><br>(Includes the following medications: zolpidem, temazepam, eszopiclone, suvorexant, triazolam, eszopiclone, ramelteon, flurazepam, zaleplon)	Risk factors for fall/fracture[b]<br><br>Therapeutic duplication or concurrent use with an opioid, benzodiazepine, muscle relaxer, or anti-convulsant<br><br>Use of a brand-name sedative/hypnotic sleeping aid
**Candidate Prescribing Practices Rated as Having Uncertain Scientific Validity**		
Statins for primary prevention of ASCVD	Use in adults **aged ≥85** without a diagnosis of atherosclerotic cardiovascular disease<br><br>**(ASCVD includes coronary artery disease, cerebrovascular disease, and peripheral arterial disease)**	~~Adults aged ≥75 without diabetes~~<br><br>~~Any adult aged ≥85 without a diagnosis of atherosclerotic cardiovascular disease~~<br><br>**Presence of a life-limiting illness**<br><br>Use of a brand-name statin

© 2022 Radomski TR et al. *JAMA Network Open.*

Inappropriate Use of Iron Supplementation	Use without an appropriate or active diagnosis (e.g., anemia or iron deficiency)	Dosing frequency greater than **daily**<br><br>History of constipation
Nitrofurantoin for the treatment or prevention of UTIs	~~Use in patients with a history of CKD~~<br><br>**Use with a diagnosis of CKD Stages 4-5 (GFR <30) or ESRD on dialysis**	~~Use with a diagnosis of CKD Stages 4-5 or ESRD on dialysis (E)~~<br><br>Chronic use >30 days<br><br>Use of brand name nitrofurantoin
Loop diuretics with a calcium channel blocker as part of a prescribing cascade	Concurrent use of a loop diuretic and a **dihydropyridine** calcium channel blocker<br><br>(Excluding patients with a history of congestive heart failure, cirrhosis, pulmonary hypertension, chronic kidney disease, and nephrotic syndrome)	New use of a loop diuretic within 6 months after receiving a new prescription for a **dihydropyridine** calcium channel blocker<br><br>~~Use of a brand-name diuretic or ethacrynic acid~~
Genitourinary anti-spasmodics in patients prescribed a cholinesterase inhibitor as part of a prescribing cascade	Concurrent use of a **non-selective** genitourinary anti-spasmodic medication (e.g., oxybutynin) and an acetylcholinesterase inhibitor	New use of a **non-selective** genitourinary anti-spasmodic medication within 6 months after receiving a new prescription for an acetylcholinesterase inhibitor<br><br>~~Use of a brand name genitourinary anti-spasmodic~~
~~Overtreatment of hypertension~~<br>Potentially unsafe use of anti-hypertensive medications	**Use of an anti-hypertensive medication from a medication class that is poorly tolerated in older adults**<br><br>**(i.e., <u>non-selective</u> alpha 1 blockers, central alpha agonists, aliskiren, minoxidil, and verapamil)**	Orthostasis or Risk factors for fall/fracture[b]<br><br>~~Use of an anti-hypertensive medication from a medication class that is poorly tolerated in older adults (i.e., alpha 1 blockers, central alpha agonists, aliskiren, minoxidil, and verapamil)~~<br><br>Use of a brand name anti-hypertensive medication

Footnotes:

[a] Bolded and stricken text indicates text that was added or removed after Rounds 1 and 2, respectively.

[b] Including age ≥80; history of cognitive disorder, prior falls/fractures, or frailty, per the claims-based algorithms contained within Green AR et al. Drugs Aging. 2019 (cognitive disorder or falls/fractures); 36: 289-297; and Kim DH et al. Gerontol. 2018; 73(7): 289-297 (Frailty)

[c] Includes patients with a history of peptic ulcer disease, other forms of upper gastrointestinal bleeding, coagulopathy, or concurrent use with other anticoagulants or NSAIDs

[d] Per the claims-based algorithm contained within Fleming-Dutra KE, et al. JAMA. 2016; 315(17): 1864-1873

<u>Abbreviations</u>: ASCVD, Atherosclerotic Cardiovascular disease, such as coronary artery disease, cerebrovascular disease, and peripheral arterial disease; PUD, peptic ulcer disease, GIB, Gastrointestinal Bleed; NSAIDs, non-steroid anti-inflammatories; TSH, Thyroid stimulating hormone; VTE, venous thromboembolism; CKD, chronic kidney disease; GERD, gastroesophageal reflux disease; COX, cyclooxygenase; PPI, proton pump inhibitor; AchE, acetylcholinesterase inhibitor; COPD, chronic obstructive pulmonary disease

© 2022 Radomski TR et al. *JAMA Network Open.*
